# Sex Differences in Time-Series Changes in Pseudo-*R*^2^ Values Regarding Hyperuricemia in Relation to the Kidney Prognosis

**DOI:** 10.3390/jpm10040248

**Published:** 2020-11-26

**Authors:** Hiroshi Kataoka, Mamiko Ohara, Toshio Mochizuki, Kazuhiro Iwadoh, Yusuke Ushio, Keiko Kawachi, Kentaro Watanabe, Saki Watanabe, Taro Akihisa, Shiho Makabe, Shun Manabe, Masayo Sato, Naomi Iwasa, Rie Yoshida, Yukako Sawara, Norio Hanafusa, Ken Tsuchiya, Kosaku Nitta

**Affiliations:** 1 Department of Nephrology, Tokyo Women’s Medical University, Tokyo 162-8666, Japan; kataoka@twmu.ac.jp (H.K.);; 2 Department of Nephrology, Clinical Research Division for Polycystic Kidney Disease, Tokyo Women’s Medical University, Tokyo 162-8666, Japan; 3 Department of Nephrology, Kameda Medical Center, Chiba 296-8602, Japan; 4 Department of Blood Purification, Tokyo Women’s Medical University, Tokyo 162-8666, Japan

**Keywords:** chronic kidney disease, hyperuricemia, prognosis, patient-centered medicine, sex difference: time-series changes in pseudo-*R*^2^

## Abstract

Studies on sex differences in time-series changes in pseudo-*R*^2^ values regarding hyperuricemia (HU) in relation to the kidney prognosis among patients with chronic kidney disease (CKD) are scant. The kidney prognosis was evaluated in 200 patients with CKD (median follow-up, 12.3 years). Survival analyses and logistic regression analyses were conducted, generating time-series pseudo-*R*^2^ values. We used four definitions of HU according to serum uric acid (SUA) levels (HU6, SUA ≥ 6.0 mg/dL; HU7, SUA ≥ 7.0 mg/dL; HU8, SUA ≥ 8.0 mg/dL) and antihyperuricemic agent use to calculate the mean and percentage of the change in pseudo-*R*^2^ values from the 6th year until the end of the study (6Y–End Mean and 6Y–End Change, respectively). The multivariable Cox regression analysis showed that HU7 was significantly associated with kidney outcomes. When stratified by sex, the 6Y–End Mean was clearly higher in women than in men for all HU definitions, with the highest value (0.1755) obtained for HU7 in women. The pseudo-*R*^2^ values for HU6 in women showed an increasing pattern, with a 6Y–End Change of 11.4%/year. Thus, it may be clinically meaningful to consider sex differences in the time-series pseudo-*R*^2^ values regarding HU and kidney outcomes.

## 1. Introduction

Although there has been long debate on the role of hyperuricemia in chronic kidney disease (CKD) progression [1,2,3,4,5,6], several recent Mendelian randomization studies showed that genetically elevated uric acid is not associated with the onset or progression of CKD [7,8]. Furthermore, two recent randomized studies in patients with type 1 diabetes (Preventing Early Renal Loss in Diabetes trial: PERL trial) and those with CKD stage 3 to 4 (Controlled Trial of Slowing of Kidney Disease Progression from the Inhibition of Xanthine Oxidase trial: CKD-FIX trial) reported that there were no clinically meaningful benefits of allopurinol on renal outcomes [9,10]. The current consensus seems to have shifted toward the idea that uric acid is not causally linked to the progression of CKD. 

However, scientists should not completely deny the possibility that uric acid does not have a negative effect on the kidneys. Indeed, a recent animal study reported that febuxostat, an urate lowering agent, reduced urinary albumin levels and alleviated glomerular, tubulointerstitial, and arteriolar injury in rats that underwent 5/6 nephrectomy and received the uricase inhibitor oxonic acid [11]. Nakagawa et al. reported that hyperuricemic rats develop glomerular hypertrophy that can be prevented, in part, by angiotensin-converting-enzyme inhibitor therapy, and concluded that prolonged hyperuricemia is associated with the development of glomerulosclerosis in rats [12]. Furthermore, it has been reported that hyperuricemia might have a causal role in the development of obesity, metabolic syndrome, and endothelial dysfunction in rats fed a high-fructose atherogenic diet [13,14]. Taken together, a direct harmful effect of uric acid on endothelial cells [12,13,15] and smooth muscle cells [11,16] in kidney arterial/arteriolar and glomerular vessels were observed in animal studies, and hyperuricemia could cause kidney arterial/arteriolar and glomerular injuries (including glomerular hypertrophy and glomerular sclerosis) and subsequent tubular interstitial injury. A similar pathophysiology of hyperuricemia has also been confirmed in humans. Namely, hyperuricemia has been reported to be associated with arterial/arteriolar nephrosclerosis in biopsy-based studies [17,18]. 

As nephrosclerosis is characterized by a slow progression of kidney dysfunction, it was previously referred as benign-nephrosclerosis. However, recently, it has been confirmed that patients with nephrosclerosis have poor long-term prognosis [19]. It has been suggested that among patients with nephrosclerosis, those with poor prognosis have accompanying glomerular hypertrophy [20]. Glomerular hypertrophy is considered to lead to glomerular sclerosis, following an absolute decrease in the number of glomeruli and resulting in kidney dysfunction [21,22,23]. Since such a decrease in the absolute number of glomeruli requires structural damage, it takes time to be expressed as a decrease in kidney function. Due to population aging, the number of patients who initiate dialysis treatment is increasing, which is considered to reflect an increase in nephrosclerosis [24]. Furthermore, kidney injury due to nephrosclerosis progresses slowly, but steadily, over the decades. Therefore, it is appropriate to set an observation period of 10 years or more to evaluate kidney outcomes due to hyperuricemia. In fact, many observational studies reporting on the relationship between hyperuricemia and kidney prognosis have evaluated long-term kidney prognosis. For example, hyperuricemia has been reported to independently predict end-stage kidney disease (ESKD) (median follow-up, 25.7 years) in persons with normal kidney function [25]. Additionally, hyperuricemia has been reported to independently predict the development of kidney disease in patients with immunoglobulin A (IgA) nephropathy (median follow-up, 10 and 11.8 years) [26,27], diabetic nephropathy (median follow-up, 18.1 years) [28], kidney transplantation (mean follow-up, 160 months) [29], and CKD (median follow-up, 10 years) [3]. Considering the results of such observational studies, it is expected that studies examining the effects of urate-lowering drugs should also be evaluated in the long term, such as more than 10 years. Although two recent randomized studies provide important data, with a high level of evidence, their follow-up period might be considered short for evaluating chronically progressive kidney disease (104 weeks in the CKD-FIX trial, 164 weeks in the PERL trial) [9,10]. As described in the limitations section of the PEAL trial [10], if urate promotes kidney damage with long-term exposure, a trial of longer duration is necessary to reveal differences between groups. In addition, the results of two recent randomized studies [9,10] may not be fully applicable to other races or ethnic groups; similarly, their results should not be generalized to patients at other stages of diabetic kidney disease and those with CKD of other stages.

Although there are many reasons for the lack of consensus regarding asymptomatic hyperuricemia in patients with CKD, we believe that it is meaningful to evaluate sex differences at various observation periods and cutoff values for serum uric acid (SUA). Considering that SUA levels are generally lower in women than in men, research regarding kidney prognoses that evaluates sex differences is desired. As there is a lack of consensus regarding hyperuricemia as a predictor for kidney outcomes, a longer observation period is important. Finally, considering that cutoff values are needed to guide the clinician in decision-making [30], research evaluating the appropriate cutoff values for various sub-cohorts is desired. We believe that an analysis of the time-series changes in pseudo-*R*^2^ values [31,32] is suitable in the context of these considerations. Furthermore, we hypothesized that the kidney pathophysiology of hyperuricemia differs between male and female patients with CKD. Therefore, we examined sex differences in the time-series changes in pseudo-*R*^2^ values in terms of kidney prognosis, using several hyperuricemia cutoff values, in patients with CKD.

## 2. Materials and Methods

### 2.1. Study Population

We reviewed the records of 2012 outpatients with CKD who visited the Kidney Center at Tokyo Women’s Medical University Hospital in Japan between August 2006 and August 2007. To assess the influence of hyperuricemia and visceral fat on the kidney prognosis, patients without abdominal computed tomography (CT) (*n* = 1811) and with nephrotic syndrome (*n* = 1) were excluded. A total of 200 patients were finally enrolled in the present study (Supplementary Figure S1 online). CKD was diagnosed according to previously described criteria [33]. The subjects’ human rights and methods for protecting their personal information were considered in detail. All relevant and responsible staff members adhered to the principles of the Helsinki Declaration (amended October 2013) and the Ethical Guidelines for Clinical Studies (revised February 28, 2017). This study was approved by the Medical Ethics Committee of Tokyo Women’s Medical University (#4189). All participants gave their written informed consent at the time of entry.

### 2.2. Covariable Assessments

Anthropometric and physical examinations, including blood pressure, body height, body weight, and visceral fat area (VFA) assessments, were conducted during a regular outpatient clinic visit. The blood pressure was measured in triplicate using a mercury sphygmomanometer; the average value was used in analyses. The VFA was measured using computed tomography. All biochemical analyses were performed on samples obtained after an overnight fast. Serum creatinine levels were measured enzymatically. The estimated glomerular filtration rate (eGFR) for Japanese patients was calculated using a previously described formula [34]. In accordance with real clinical settings, hyperuricemia was defined using four different criteria as follows: HU6, SUA ≥ 6.0 mg/dL or antihyperuricemic agent use; HU7, SUA ≥ 7.0 mg/dL or antihyperuricemic agent use; HU8, SUA ≥ 8.0 mg/dL or antihyperuricemic agent use; and HU (Drug), antihyperuricemic agent use. Concomitant drug use (antihypertensive drugs and diuretics and drugs for the treatment of hyperuricemia, dyslipidemia, and diabetes mellitus) and comorbidities at entry were also assessed [35,36]. Hypertension was defined as systolic blood pressure ≥ 140 mmHg, diastolic blood pressure ≥ 90 mmHg, or antihypertensive agent use. Hyperglycemia was defined as a blood glucose level ≥ 110 mg/dL. Diabetes mellitus was defined as a glycated hemoglobin level ≥ 6.5%, a diagnosis of diabetes mellitus, or antidiabetic agent use. Hypertriglyceridemia was defined as a serum triglyceride level ≥ 150 mg/dL or oral lipid-lowering agent use. Hypercholesterolemia was defined as a serum total cholesterol level ≥ 220 mg/dL, serum low-density lipoprotein cholesterol level ≥ 140 mg/dL, or oral lipid-lowering agent use. Low high-density lipoprotein (HDL) cholesterol was defined as a serum HDL cholesterol level  ≤  40 mg/dL. Diabetic kidney disease, chronic glomerulonephritis, and nephrosclerosis were diagnosed either from biopsies or clinically by the doctor in charge. The participants were followed up until December 31, 2019.

### 2.3. Study End Point

The study’s end point was kidney disease progression, which was defined as a ≥30% decrease in the eGFR from baseline (≥30% eGFR decrease) or ESKD development requiring dialysis [37].

### 2.4. Statistical Analysis

Continuous variables are expressed as means and standard deviations (SDs) or as medians (quartile 1, quartile 3). Categorical variables are expressed as percentages, unless otherwise stated. Group differences were evaluated using unpaired Student’s *t*-test, Mann-Whitney U test, chi-squared test, or Fisher’s exact test, as appropriate. Logistic regression analyses were used to generate the McFadden pseudo-*R*-squared (pseudo-*R*^2^) values as a measure of the goodness of fit of the kidney prognostic models [38]. The annual percentage of change of pseudo-*R*^2^ values (%/year) was calculated as follows: % pseudo-*R*^2^ change = pseudo-*R*^2^ slope/baseline pseudo-*R*^2^ × 100. The pseudo-*R*^2^ slope was calculated by the least squares method. Kidney prognostic factors were also evaluated in Cox regression analyses, and the Kaplan–Meier method was used for survival analyses. Interactions between sex and each variable of interest were considered by adding sex and the interaction term to the multivariable Cox proportional hazards models. Analyses were conducted using the entire cohort and a sub-cohort of patients without antihyperuricemic agent use, with and without stratification by sex. *P*-values < 0.05 were considered statistically significant. All statistical analyses were performed using JMP Pro software for Windows version 15.0.0 (SAS Institute, Cary, NC1, USA).

## 3. Results

### 3.1. Patient Characteristics

Table 1 presents the baseline characteristics of the entire cohort, overall and according to sex. Comparative analyses revealed that the MBP, body mass index, VFA, SUA level, urine albumin-to-creatinine ratio, and proportion of patients with VFA ≥ 100 cm^2^ were higher and the eGFR was lower in men than in women. Regarding the primary causes of CKD, concomitant drug administration, comorbidities, the rates of nephrosclerosis, antihypertensive agent use, hypertension, hyperuricemia, hypertriglyceridemia, and hyperglycemia were higher and the rate of chronic glomerulonephritis was lower in men than in women. Supplementary Table S1 online presents the baseline characteristics in the sub-cohort of patients without antihyperuricemic agent use.

### 3.2. Hyperuricemia as a Progression-Related Factor in Patients with Chronic Kidney Disease

The median follow-up duration was 12.3 years (interquartile range: 5.9–12.9 years), during which 8 patients (7 men, 1 woman) died, 41 (29 men, 12 women) reached ESKD, and 84 (48 men, 36 women) reached the primary endpoint (i.e., ≥ 30% eGFR decline or ESKD). The results of the univariable and multivariable Cox regression analyses of the entire cohort are provided in Table 2. The multivariable Cox regression analysis of the entire cohort showed that the eGFR, urine albumin-to-creatinine ratio, hyperglycemia, and HU7 were independent risk factors for CKD progression; however, HU6 and HU8 did not reach statistical independence. Interactions between sex and the examined variables in terms of the risk of CKD progression were not significant. The results of the multivariable Cox regression analyses stratified by sex as well as in the sub-cohort of patients without antihyperuricemic agent use are provided in Supplementary Table S2 online. Among men, disease progression was not significantly associated with hyperuricemia, by any definition. Among women, disease progression was significantly associated with HU7. Among patients without antihyperuricemic agents, disease progression was significantly associated with HU7.

The Kaplan–Meier analysis showed that the kidney survival rate was significantly lower in patients with HU6, HU7, and HU8 than in those without HU6, HU7, and HU8, respectively, in all cohorts (Supplementary Figure S2 online).

### 3.3. Time-Series Changes in Pseudo-R^2^ Values in Terms of the Prognostic Efficacy

**Figure 1:**
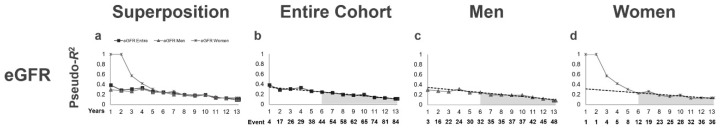
Time-series changes in pseudo-*R*^2^ values for the kidney outcome in terms of the eGFR. (**a**) Pseudo-*R*^2^ values for all cohorts are superimposed. The lines represent the time-series changes in pseudo-*R*^2^ values for the kidney outcome in terms of the eGFR for the entire cohort, men, and women (shown individually in **b**–**d**). Pseudo-*R*^2^ values for the entire cohort (**b**), men (**c**), and women (**d**). Dotted lines represent the least-squares regression line between the 6th year and the study end. The gray area under the lines represents the 6Y–End Mean. Abbreviations: eGFR, estimated glomerular filtration rate; 6Y–End Mean, mean value of pseudo-*R*^2^ values from the 6th year to the end of the observation period.

Initially, we examined the pseudo-*R*^2^ values for the eGFR, which is an established potent risk factor of CKD progression (Table 3, Figure 1). As shown in Figure 1, the pseudo-*R*^2^ values for the eGFR in the entire cohort gradually decreased over time. Additionally, the pseudo-*R*^2^ values for the eGFR were significantly higher in women than in men during years 1 through 5, but were comparable from year 6 (when the number of events reached 12) until the end of the study. Accordingly, we determined that the pseudo-*R*^2^ values after the 6th year were reliable and universal. Thus, we calculated the mean value and percentage of change of pseudo-*R*^2^ values for the 6th year to the end of the observation period (6Y–End Mean and 6Y-End Change, respectively) (Table 3).

**Figure 2:**
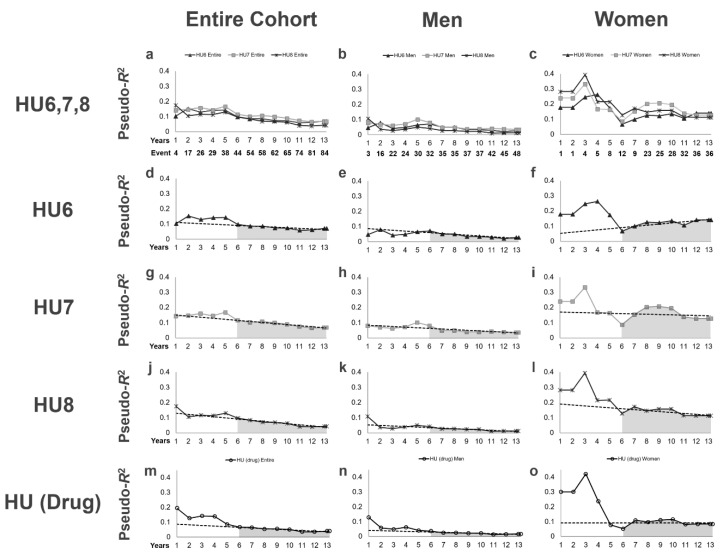
Time-series changes in pseudo-*R*^2^ values for the kidney outcome in terms of each type of hyperuricemia. (**a**–**c**) Pseudo-*R*^2^ values in all definitions of hyperuricemia for the entire sub-cohort and for each sex are superimposed. The lines represent the time-series changes in pseudo-*R*^2^ values for the kidney outcome in terms of each definition of hyperuricemia for the entire cohort (**a**), men (**b**), and women (**c**). (**d**–**f**) Pseudo-*R*^2^ values in terms of HU6. (**g**–**i**) Pseudo-*R*^2^ values in terms of HU7. (**j**–**l**) Pseudo-*R*^2^ values in terms of HU8. (**m**–**o**) Pseudo-*R*^2^ values in terms of HU (Drug). Dotted lines, least-squares regression line from the 6th year to the study end; gray area under the lines, 6Y–End Mean. Abbreviations: HU6, serum uric acid level ≥ 6.0 mg/dL or antihyperuricemic agent use; HU7, serum uric acid level ≥ 7.0 mg/dL or antihyperuricemic agent use; HU8, serum uric acid level ≥ 8.0 mg/dL or antihyperuricemic agent use; HU (Drug), antihyperuricemic agent use; 6Y–End Mean, mean pseudo-*R*^2^ values from the 6th year to the end of the observation period.

We then examined the pseudo-*R*^2^ values for kidney outcomes in terms of each definition of hyperuricemia (Table 4, Figure 2). In patients with HU6, 7 patients died, 38 reached ESKD and 66 reached the primary endpoint during the follow-up period. In patients with HU7, 5 patients died, 36 reached ESKD, and 57 reached the primary endpoint during the follow-up period. In patients with HU8, 5 patients died, 31 reached ESKD, and 48 reached the primary endpoint during the follow-up period. In patients with HU (Drug), 4 patients died, 28 reached ESKD, and 44 reached the primary endpoint during the follow-up period. For all definitions of hyperuricemia, the pseudo-*R*^2^ values in the entire cohort gradually decreased over time. Furthermore, among the four definitions of hyperuricemia, the highest 6Y–End Mean (0.0976) was obtained for HU7. When stratified by sex, the 6Y–End Mean was visibly higher in women than in men for all definitions of hyperuricemia, as shown by the grey area under the lines in Figure 2. The highest 6Y–End Mean (0.1755) was obtained for HU7 in women (Table 4, Figure 2i). Interestingly, the pseudo-*R*^2^ values for HU6 in women showed an increasing pattern after 6 years, with a 6Y–End Change of 11.4%/year, and increased to their highest value of 0.1403 at the end of the follow-up period (Table 4, Figure 2f), during which the pseudo-*R*^2^ values for HU6 were even higher than those for HU7. Furthermore, the 6Y–End Mean for HU (Drug) was higher in women than in men and showed a stable pattern, with a 6Y–End Change of -0.0%/year (Table 4, Figure 2o), suggesting that women who received antihyperuricemic agents have a poor renal prognosis.

**Figure 3:**
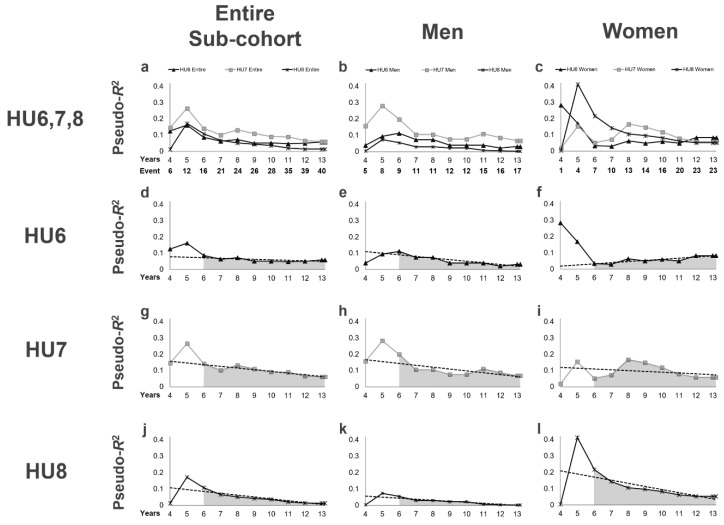
Time-series changes in pseudo-*R*^2^ values for the kidney outcome in terms of each definition of hyperuricemia in the sub-cohort of patients without antihyperuricemic agent use. (**a**–**c**) Pseudo-*R*^2^ values in all definitions of hyperuricemia for the entire sub-cohort and for each sex are superimposed. The lines represent the time-series changes in pseudo-*R*^2^ values for the kidney outcome in terms of each definition of hyperuricemia for the entire sub-cohort (**a**), men (**b**), and women (**c**). (**d**–**f**) Pseudo-*R*^2^ values in terms of HU6. (**g**–**i**) Pseudo-*R*^2^ values in terms of HU7. (**j**–**l**) Pseudo-*R*^2^ values in terms of HU8. Dotted lines, least-squares regression line from the 6th year to the study end; gray area under the lines, 6Y–End Mean. Abbreviations: HU6, serum uric acid level ≥ 6.0 mg/dL or antihyperuricemic agent use; HU7, serum uric acid level ≥ 7.0 mg/dL or antihyperuricemic agent use; HU8, serum uric acid level ≥8.0 mg/dL or antihyperuricemic agent use; 6Y–End Mean, mean pseudo-*R*^2^ values from the 6th year to the end of the observation period.

Third, we also examined the pseudo-*R*^2^ values for kidney outcomes in the sub-cohort of patients without antihyperuricemic agent use (Table 5, Figure 3). In this sub-cohort, 4 patients (3 men, 1 woman) died, 13 (9 men, 4 women) reached ESKD, and 40 (17 men, 23 women) reached the primary endpoint during follow-up period. As shown in Figure 3, we confirmed that the results for the sub-cohort were similar to those for the entire cohort. For each definition of hyperuricemia, the pseudo-*R*^2^ values in this sub-cohort gradually decreased over time, and the 6Y–End Mean was the highest for HU7, which was confirmed even in men (Table 5, Figure 3h). Additionally, similar to that for the entire cohort, the pseudo-*R*^2^ values for HU6 in women showed an increasing pattern after 6 years and increased to their highest value (0.0833) at the end of the follow-up period (Table 5, Figure 3f).

## 4. Discussion

Patient-centered medicine has recently attracted increased attention [39,40]. In patient-centered medicine, it is necessary to treat individual patients according to their heterogeneous characteristics [41]. Thus, the development of patient-centered medicine requires the disaggregation of data and analyses of differences in sub-cohorts [42,43]. Considering that the treatment of patients in real clinical settings requires multifaceted and comprehensive judgments based on abundant medical information, it is meaningful to examine various cutoff values in various sub-cohorts, at various follow-up periods, especially for important prognostic factors. Indeed, the appropriate cutoff value for a risk factor can depend on the handling of the risk factor [43,44,45], the cohort evaluated [43,44,46,47], and the observation period [31,32]. 

The present study aimed to elucidate the significance of time-series changes in pseudo-*R*^2^ values in terms of hyperuricemia as kidney disease progression in male and female patients with CKD. Regarding hyperuricemia, the cutoff value for SUA in terms of the kidney prognosis must be evaluated from various perspectives such as sex differences or observation-period differences. To clarify the differences between male and female patients with CKD in terms of hyperuricemia, we examined the efficacy of not only three different hyperuricemia cutoff points in the entire cohort but also in men and women separately. The present study is the first to demonstrate the time-series changes in pseudo-*R*^2^ values for the eGFR as well as for hyperuricemia defined by several cutoff points in terms of their kidney prognostic abilities in patients with CKD. The important results of the present study are as follows. First, we confirmed that HU7 was significantly associated with the kidney prognosis in patients with CKD using multivariable Cox analysis. Second, we found that the time-series changes in pseudo-*R*^2^ values for hyperuricemia in kidney disease progression were higher in women than in men. Third, and most importantly, we found that the time-series changes in pseudo-*R*^2^ values for HU6 in terms of kidney disease progression in women increased over time.

Evaluating the time-series changes in pseudo-*R*^2^ values is one of the clinical approaches we have recently adopted, based on the fact that the prognostic abilities of clinical risk factors vary with time [31,32]. Basically, variables that maintain a high pseudo-*R*^2^ value during follow-up or show an increasing pattern in pseudo-*R*^2^ values over time are considered to reflect the long-term prognosis and are important prognostic factors. Meanwhile, variables that show an acute decreasing pattern in pseudo-*R*^2^ values over time can be used to predict the short-term prognosis or to evaluate the therapeutic reactivity [31]. The coexistence of short- and long-term kidney prognostic factors in the real world means that the best kidney prognostic factors determined by a multivariable Cox analysis can change depending on the length of the follow-up observation period. Therefore, awareness of the relationship between the time-series changes in pseudo-*R*^2^ values and the prognostic ability of a risk factor is clinically meaningful. Indeed, in our previous study [31], some kidney risk factors, such as the Oxford S and T scores in IgA nephropathy (which have been established as kidney prognostic factors in IgA nephropathy), showed increased pseudo-*R*^2^ values over time. Evaluation of the time-series changes in pseudo-*R*^2^ values is useful because the effect of the observation period on the prognostic ability of a variable over time can be visually presented. In a further refinement, the present study examined the time-series changes in pseudo-*R*^2^ values for one variable (hyperuricemia) using different cutoff values. The results showed that the pseudo-*R*^2^ values for the eGFR were nearly the same in men and women from the 6th year to the end of the study, indicating the reliability of pseudo-*R*^2^ values as a tool for kidney outcome evaluation.

Among the various definitions of hyperuricemia used in the present study, the highest 6Y–End Mean was obtained for HU7 in the entire cohort and sex-based sub-cohorts as well as in the sub-cohort of patients without antihyperuricemic agent use. These results may imply a strong link between the long-term kidney prognosis and hyperuricemia defined by an SUA level ≥ 7.0 mg/dL. The solubility of uric acid is affected by temperature, pH, mechanical stress, and serum factors [48]. Under physiological conditions, urate spontaneously crystallizes at concentrations > 6.8 mg/dL of uric acid, and the deposition of monosodium urate crystals activates tissue macrophages, which secrete inflammatory cytokines [49]. Based on these characteristics, an SUA level > 7.0 mg/dL is generally accepted as the reference for hyperuricemia [50]. Although no pathological examination was performed in the present study, considering that the development of ESKD was common in patients with gout prior to the availability of urate-lowering therapies [51], we do not deny that urate crystals may adversely affect the kidney prognosis.

Meanwhile, the 6Y–End Mean was higher in women than in men for all definitions of hyperuricemia in the entire cohort. This result may indicate that the association between hyperuricemia and the kidney prognosis is stronger in women than in men and that it is better to evaluate the effects of hyperuricemia on the kidney prognosis in women than in men. Interestingly, in women, the highest pseudo-*R*^2^ value among the various definitions of hyperuricemia clearly shifted toward a lower cutoff as the observation period was extended (Table 4, gray area): from HU (Drug) < HU6 < HU7 < HU8 at year 6 to HU (Drug) < HU8 < HU7 < HU6 at the study end. This result indicates that higher cutoff values are useful for the short-term prognosis, whereas lower cutoff values are useful for the long-term prognosis. For cutoff values that are not clinically meaningful, the pseudo-*R*^2^ values do not increase, even if the observation period is extended. In the present study, the pseudo-*R*^2^ values for HU6 in women showed an increasing pattern after 6 years and increased to 0.1403 at the end of the follow-up period, which was the highest value among all definitions of hyperuricemia. Although HU6 did not show a statistically significant difference as a kidney prognostic factor in the multivariable Cox analysis in the present study, the observed time-series changes in pseudo-*R*^2^ values for HU6 in women imply that this variable would become a long-term kidney prognostic factor in women by a further extension of the observation period. Additionally, the positive results regarding HU6 implies the existence of an underlying pathophysiological mechanism other than urate crystals for the relationship between hyperuricemia and the kidney prognosis. Consistent with this, in 2002, Kang et al. reported that, in laboratory animals with CKD and hyperuricemia, the kidney disease progressed rapidly even without crystals in the kidney [52]. Subsequently, various pathological conditions for the involvement of hyperuricemia in the kidney prognosis have been advocated, and recent data have shown a direct harmful effect of SUA on endothelial cells [13,15] and smooth muscle cells [16,53]. Hyperuricemia has also been found to induce systemic hypertension and afferent arteriolar sclerosis in animal models [12,54], and high SUA levels have been reported to be associated with kidney arteriolosclerosis in humans [17,18]. Especially, the kidney biopsy study by Kohagura et al. [17] reported lower cutoff values for the SUA level in women than in men, in terms of the risk for arteriolar hyalinosis or arteriolar wall thickening.

Epidemiologically, women generally have lower SUA levels, and estrogen is considered to be associated with lower SUA levels [55]. Estrogen suppresses the protein levels of urate reabsorption transporter 1, which increases uric acid excretion in the kidney, resulting in a decreased SUA level [56]. The presence of estrogen is believed to protect women against kidney injury [57]. Indeed, men have substantially higher CKD and ESKD rates than women [58,59,60]. Furthermore, it has recently been reported that sex differences in hyperuricemia affect the kidney prognosis [61,62]. Nagasawa et al. [61] reported an elevated SUA level as an independent risk factor for the progression of IgA nephropathy in female patients but not in male patients. Akasaka et al. [62] reported that women are more susceptible to urate-induced yearly decrease in the eGFR than men. Although the pathophysiological mechanism of the sex difference in the association between the SUA level and CKD progression remains unclear, we consider the relative balance of kidney prognostic factors between sub-cohorts to be related to this sex difference. In the Chronic Kidney Disease Japan Cohort study [43], differences in the numbers of kidney prognostic factors were reported, with fewer kidney prognostic factors in patients with earlier stages of CKD than in those with later stages of CKD, and fewer kidney prognostic factors in women than in men. The relatively small number of kidney prognostic factors in women could increase the relative importance of each factor. Just as relatively highly progressed stages of CKD may mask the effect of hyperuricemia regarding the kidney prognosis [51], women, with fewer kidney prognostic factors compared to those in men, may be more affected by hyperuricemia than are men.

The present study has several limitations. First, the serum creatinine level was based on a single assessment at baseline, which may have been influenced by existing comorbidities at the time of the assessment. Second, the association between hyperuricemia and kidney outcomes may not be generalizable to other populations, as all the participants were Japanese. Third, the impact of subsequent changes in hyperuricemia on outcomes was difficult to demonstrate because only baseline laboratory data were used in the analyses. Fourth, based on the consideration that hyperuricemia is associated with metabolic syndrome [63], the present study excluded 90% of the patients without CT scan data for the assessment of abdominal fat. Due to this exclusion, potential selection bias was unavoidable because the patients who underwent CT examination voluntarily enrolled in this study. Fifth, the male participants had lower kidney function and higher proportion of hyperuricemia than the female participants. This may have influenced the kidney prognosis analyses; thus, the present study alone cannot rule out an association between hyperuricemia and a poor kidney prognosis in men. Further studies with larger cohorts are expected. However, the present study has several strengths. First, this is the first study to evaluate the time-series changes in pseudo-*R*^2^ values for the eGFR and hyperuricemia at several cutoff points. Second, the present study utilized a well-characterized population of Japanese patients with CKD who were treated by nephrologists at a single center using standard CKD care guidelines. Third, the detailed analyses were designed to disaggregate the data using time-series analyses based on several definitions of hyperuricemia (with different cutoff values) stratified by sex, which is important for achieving patient-centered medicine [39,40].

## 5. Conclusions

We confirmed that HU7 is significantly associated with the kidney prognosis in patients with CKD. The time-series pseudo-*R*^2^ values for hyperuricemia as kidney disease progression were higher in women than in men, and the time-series changes in pseudo-*R*^2^ values for HU6 as kidney disease progression in women increased over time. It may be clinically meaningful to consider sex differences in the time-series pseudo-*R*^2^ values for hyperuricemia in terms of the kidney outcome.

## Figures and Tables

**Table 1:** Patient characteristics according to sex (*n* = 200).

Variables	Entire Cohort	Men	Women	*P*-Value
	*n* = 200	*n* = 107	*n* = 93	
*Clinical and Laboratory Findings*				
Age (years)	59.2 ± 12.8 [200]	59.7 ± 12.9	58.6 ± 12.8	0.5448
Sex (Men; %)	107 (53.5) [200]	107 (100.0)	0 (0.0)	<0.0001
MBP (mmHg)	92.6 ± 6.3 [200]	93.4 ± 6.3	91.7 ± 6.3	0.0491
BMI (kg/m^2^)	24.0 ± 3.9 [200]	24.6 ± 3.4	23.4 ± 4.3	0.0342
Visceral fat area (cm^2^)	126.9 ± 61.4 [200]	150.1 ± 60.5	100.1 ± 50.8	<0.0001
Visceral fat area 100 cm^2^ (vs. no)	129 (64.5) [200]	86 (80.4)	43 (46.2)	<0.0001
eGFR (mL/min/1.73m^2^)	56.0 ± 22.4 [200]	52.8 ± 22.5	59.7 ± 21.9	0.0299
Uric Acid (mg/dL)	5.83 ± 1.50 [199]	6.42 ± 1.27	5.14 ± 1.45	<0.0001
UACR (mg/g Cre)	66.2 (22.3–252.5) [200]	90.2 (26.0–860.2)	50.9 (21.1–115.6)	0.0153
*Primary cause of CKD*				
Diabetic nephropathy (%)	18 (9.0) [200]	12 (11.2)	6 (6.5)	0.3233
Chronic glomerulonephritis (%)	104 (52.0) [200]	47 (43.9)	57 (61.3)	0.0142
Nephrosclerosis (%)	41 (20.5) [200]	33 (30.8)	8 (8.6)	0.0001
Others (%)	37 (18.5) [200]	15 (14.0)	22 (23.7)	0.0800
*Concomitant drugs*				
Antihypertensive agents (%)	140 (70.0) [200]	84 (78.5)	56 (60.2)	0.0049
ARB and or ACEI	113 (56.5) [200]	72 (67.3)	41 (44.1)	0.0010
CCB	62 (31.0) [200]	34 (31.8)	28 (30.1)	0.7992
Antidiabetic agents (%)	26 (13.0) [200]	17 (15.9)	9 (9.7)	0.1927
Corticosteroids (%)	28 (14.0) [200]	16 (15.0)	12 (12.9)	0.6769
Immunosuppressants (%)	13 (6.5) [200]	8 (7.5)	5 (5.4)	0.5811
Diuretics (%)	51 (25.5) [200]	25 (23.4)	26 (28.0)	0.4573
*Comorbidities*				
Hypertension (%)	139 (69.5) [200]	83 (77.6)	56 (60.2)	0.0078
HU6 (%)	122 (61.0) [200]	87 (81.3)	35 (37.6)	<0.0001
HU7 (%)	100 (50.0) [200]	75 (70.1)	25 (26.9)	<0.0001
HU8 (%)	86 (43.0) [200]	66 (61.7)	20 (21.5)	<0.0001
HU (Drug) (%)	78 (39.0) [200]	60 (56.1)	18 (19.4)	<0.0001
Hypertriglyceridemia (%)	120 (60.0) [200]	71 (66.4)	49 (52.7)	0.0491
Hypercholesterolemia (%)	123 (61.5) [200]	63 (58.9)	60 (64.5)	0.4138
Low HDL cholesterol (%)	93 (46.5) [200]	56 (52.3)	37 (39.8)	0.0759
Hyperglycemia (%)	66 (33.0) [200]	43 (40.2)	23 (24.7)	0.0204

Continuous variables are expressed as means and standard deviations or as medians (quartile 1–quartile 3). Categorical variables are expressed as *n* (%). Values of non-missing data are shown in brackets. Abbreviations: *P*, calculated probability; MBP, mean blood pressure; BMI, body mass index; eGFR, estimated glomerular filtration rate; UACR, urine albumin-to-creatinine ratio; Cre, creatinine; CKD, chronic kidney disease; ARB, angiotensin II receptor blocker; ACEI, angiotensin-converting enzyme inhibitor; CCB, calcium-channel blocker; HU6, serum uric acid level ≥6.0 mg/dL or antihyperuricemic agent use; HU7, serum uric acid level ≥7.0 mg/dL or antihyperuricemic agent use; HU8, serum uric acid level ≥8.0 mg/dL or antihyperuricemic agent use; HU (Drug), antihyperuricemic agent use; HDL, high-density lipoprotein.

**Table 2:** Results of the univariable and multivariable analyses for the risk factors associated with kidney outcome (i.e., a ≥30% estimated glomerular filtration rate decrease or end-stage kidney disease) among the entire study population (*n* = 200).

Variables	Univariable Analysis		Multivariable Analysis for HU6			Multivariable Analysis for HU7		
	Hazard Ratio (95% CI)	*P*-Value	Hazard Ratio (95% CI)	*P*-Value	*P*-INT	Hazard Ratio (95% CI)	*P*-Value	*P*-INT
Age (1-year increments)	1.02 (1.00–1.04)	0.0440	0.99 (0.97–1.01)	0.4726	0.6684	0.99 (0.97–1.01)	0.4531	0.6870
Men (vs. women)	1.45 (0.95–2.25)	0.0881	0.74 (0.42–1.29)	0.2857	-	0.70 (0.40–1.22)	0.2028	-
eGFR (10-mL/min/1.73 m^2^ increments)	0.63 (0.55–0.71)	<0.0001	0.70 (0.60–0.81)	<0.0001	0.2813	0.71 (0.61–0.82)	<0.0001	0.2304
UACR (10-mg/g Cre increments)	1.00 (1.00–1.01)	<0.0001	1.00 (1.00–1.01)	0.0011	-	1.00 (1.00–1.01)	0.0059	-
HU6 (vs. no)	3.31 (2.01–5.76)	<0.0001	1.68 (0.86–3.28)	0.1259	0.4985	-	-	-
HU7 (vs. no)	3.28 (2.09–5.27)	<0.0001	-	-	-	1.78 (1.00–3.16)	0.0457	0.3700
HU8 (vs. no)	2.64 (1.72–4.10)	<0.0001	-	-	-	-	-	-
HU (Drug) (vs. no)	2.45 (1.59–3.77)	<0.0001	-	-	-	-	-	-
Low HDL cholesterol (vs. no)	1.76 (1.15–2.74)	0.0097	1.12 (0.68–1.83)	0.6646	0.5443	1.13 (0.69–1.87)	0.6219	0.6391
Hypertension (vs. no)	2.16 (1.30–3.79)	0.0024	1.33 (0.74–2.37)	0.3404	-	1.35 (0.76–2.41)	0.2943	-
Hyperglycemia (vs. no)	1.95 (1.25–3.01)	0.0035	1.78 (1.11–2.87)	0.0171	-	1.75 (1.09–2.81)	0.0228	-
Visceral fat area 100 cm^2^ (vs. no)	2.15 (1.34–3.57)	0.0012	1.43 (0.77–2.68)	0.2581	0.5757	1.49 (0.79–2.80)	0.2086	0.4503

Variables with a *P*-value < 0.1 in the univariable model as well as age, sex, eGFR, and hyperuricemia were included in the multivariable model. The *P*-value for interaction is the interaction between sex and each variable of interest for the kidney outcome in the multivariable analysis. Abbreviations: CI, confidence interval; *P*, calculated probability; *P*-INT, *P*-value for interaction; eGFR, estimated glomerular filtration rate; UACR, urine albumin-to-creatinine ratio; HU6, serum uric acid level ≥ 6.0 mg/dL or antihyperuricemic agent use; HU7, serum uric acid level ≥ 7.0 mg/dL or antihyperuricemic agent use; HU8, serum uric acid level ≥ 8.0 mg/dL or antihyperuricemic agent use; HU (Drug), antihyperuricemic agent use; HDL, high-density lipoprotein.

**Table 3:** Time-series change in pseudo-*R*^2^ values of the prognostic efficacy for kidney outcomes: eGFR (*n* = 200).

	Entire Cohort	Men	Women
Years/Period	eGFR	eGFR	eGFR
1Y	0.3891	0.2974	1.0000
2Y	0.2882	0.2762	1.0000
3Y	0.3065	0.2613	0.5731
4Y	0.3314	0.3146	0.4192
5Y	0.2626	0.2439	0.3067
6Y	0.2470	0.2468	0.2268
7Y	0.2315	0.1986	0.2638
8Y	0.1978	0.1986	0.1857
9Y	0.1821	0.1937	0.1585
10Y	0.1945	0.1937	0.1935
11Y	0.1388	0.1504	0.1227
12Y	0.1283	0.1226	0.1351
13Y	0.1116	0.0899	0.1351
END	0.1116	0.0899	0.1351
1–5Y Mean	0.3130	0.2807	0.6614
6Y–End Mean	0.1897	0.1830	0.1913
6Y–End Change (%/year)	−7.7	−8.1	−7.0

Abbreviations: eGFR, estimated glomerular filtration rate; Y, years; END, study end; 1–5Y Mean, mean value of pseudo-*R*^2^ during years 1 to 5; 6Y–End Mean, mean value of pseudo-*R*^2^ from the 6th year to the study end; 6Y–End Change, percentage of change of pseudo-*R*^2^ values from the 6th year to the study end.

**Table 4:** Time-series change in pseudo-*R*^2^ values of the prognostic efficacy for kidney outcomes regarding hyperuricemia in the entire cohort (*n* = 200).

	Entire Cohort (*n* = 200)				Men (*n* = 107)				Women (*n* = 93)			
Years/Period	HU6	HU7	HU8	HU (Drug)	HU6	HU7	HU8	HU (Drug)	HU6	HU7	HU8	HU (Drug)
1Y	0.1022	0.1435	0.1749	**0.1954**	0.0461	0.0793	0.1079	**0.1293**	0.1784	0.2404	0.2817	**0.3013**
2Y	**0.1531**	0.1478	0.1046	0.1268	**0.0802**	0.0701	0.0368	0.0582	0.1784	0.2404	0.2817	**0.3013**
3Y	0.1301	**0.1600**	0.1158	0.1420	0.0425	**0.0623**	0.0277	0.0492	0.2461	0.3338	0.3934	**0.4221**
4Y	0.1415	**0.1450**	0.1128	0.1396	0.0480	**0.0718**	0.0375	0.0626	**0.2631**	0.1681	0.2150	0.2379
5Y	0.1433	**0.1681**	0.1302	0.0862	0.0652	**0.1016**	0.0497	0.0411	0.1725	0.1639	**0.2159**	0.0777
6Y	0.0983	**0.1154**	0.0973	0.0670	0.0712	**0.0809**	0.0421	0.0365	0.0669	0.0860	**0.1282**	0.0521
7Y	0.0859	**0.1023**	0.0846	0.0642	**0.0501**	0.0487	0.0267	0.0249	0.0999	0.1537	**0.1718**	0.1096
8Y	0.0859	**0.1078**	0.0702	0.0541	**0.0501**	0.0487	0.0267	0.0249	0.1255	**0.2031**	0.1464	0.0975
9Y	0.0741	**0.1003**	0.0686	0.0548	0.0337	**0.0391**	0.0227	0.0223	0.1223	**0.2073**	0.1582	0.1109
10Y	0.0723	**0.0895**	0.0629	0.0510	0.0337	**0.0391**	0.0227	0.0223	0.1359	**0.1956**	0.1581	0.1153
11Y	0.0591	**0.0749**	0.0415	0.0350	0.0288	**0.0425**	0.0112	0.0133	0.1068	**0.1389**	0.1148	0.0821
12Y	0.0620	**0.0658**	0.0392	0.0349	0.0212	**0.0390**	0.0118	0.0153	**0.1403**	0.1281	0.1131	0.0839
13Y	**0.0718**	0.0691	0.0436	0.0401	0.0279	**0.0362**	0.0126	0.0175	**0.1403**	0.1281	0.1131	0.0839
End	**0.0718**	0.0691	0.0436	0.0401	0.0279	**0.0362**	0.0126	0.0175	**0.1403**	0.1281	0.1131	0.0839
1–5Y Mean	0.1369	**0.1522**	0.1214	0.1373	0.0566	**0.0737**	0.0452	0.0638	0.2158	0.2361	0.2847	**0.2877**
6Y–End Mean	0.0829	**0.0976**	0.0669	0.0539	0.0410	**0.0489**	0.0225	0.0233	0.1355	**0.1755**	0.1525	0.1045
6Y–End Change (%/year)	**−3.9**	−6.1	−7.8	−6.4	−7.6	**−5.1**	−8.6	−6.2	**11.4**	−2.3	−4.7	−0.0

The pseudo-*R*^2^ values in bold are the highest values among the various hyperuricemia definitions within the same year. The 1–5Y Mean, 6Y–End Mean, and 6Y–End Change values in bold are the highest values among the various hyperuricemia definitions within the same period. Abbreviations: HU6, serum uric acid ≥6.0 mg/dL or taking an antihyperuricemic agent; HU7, serum uric acid level ≥7.0 mg/dL or taking an antihyperuricemic agent; HU8, serum uric acid level ≥8.0 mg/dL or taking an antihyperuricemic agent; HU (Drug), taking an antihyperuricemic agent; Y, years; 1–5Y Mean, mean value of pseudo-*R*^2^ during years 1 to 5; 6Y–End Mean, mean value of pseudo-*R*^2^ from the 6th year to the study end; 6Y–End Change, percentage of change of pseudo-*R*^2^ values from the 6th year to the study end.

**Table 5:** Time-series change in pseudo-*R*^2^ values of the prognostic efficacy for kidney outcomes regarding hyperuricemia in the sub-cohort without taking antihyperuricemic agents (*n* = 122).

	Entire Sub-cohort (*n* = 122)			Men (*n* = 47)			Women (*n* = 75)		
Years/Period	HU6	HU7	HU8	HU6	HU7	HU8	HU6	HU7	HU8
4Y	0.1252	**0.1468**	0.0128	0.0396	**0.1577**	0.0034	**0.2839**	0.0186	0.0051
5Y	0.1615	**0.2647**	0.1731	0.0942	**0.2826**	0.0742	0.1692	0.1540	**0.4130**
6Y	0.0863	**0.1417**	0.1087	0.1122	**0.1992**	0.0551	0.0338	0.0522	**0.2164**
7Y	0.0630	**0.1026**	0.0671	0.0737	**0.1051**	0.0304	0.0301	0.0723	**0.1432**
8Y	0.0721	**0.1323**	0.0517	0.0737	**0.1051**	0.0304	0.0632	**0.1667**	0.1053
9Y	0.0512	**0.1098**	0.0437	0.0397	**0.0764**	0.0222	0.0497	**0.1478**	0.0964
10Y	0.0516	**0.0914**	0.0371	0.0397	**0.0764**	0.0222	0.0598	**0.1181**	0.0821
11Y	0.0472	**0.0898**	0.0211	0.0399	**0.1100**	0.0074	0.0499	**0.0788**	0.0625
12Y	0.0505	**0.0668**	0.0151	0.0214	**0.0869**	0.0046	**0.0833**	0.0594	0.0525
13Y	0.0589	**0.0620**	0.0138	0.0313	**0.0679**	0.0025	**0.0833**	0.0594	0.0525
End	0.0589	**0.0620**	0.0138	0.0313	**0.0679**	0.0025	**0.0833**	0.0594	0.0525
6Y–End Mean	0.0650	**0.1052**	0.0432	0.0544	**0.1059**	0.0207	0.0665	**0.1055**	0.1014
6Y–End Change (%/year)	**−3.6**	−7.4	−10.4	−8.9	**−5.7**	−11.6	**20.7**	−9.5	−8.8

The pseudo-*R*^2^ values in bold are the highest values among the various hyperuricemia definitions within the same year. The values of 6Y–End Mean and 6Y–End Change in bold are the highest values among the various hyperuricemia definitions within the same period. Abbreviations: HU6, serum uric acid level ≥ 6.0 mg/dL or taking an antihyperuricemic agent; HU7, serum uric acid level ≥ 7.0 mg/dL or taking an antihyperuricemic agent; HU8, serum uric acid level ≥ 8.0 mg/dL or taking an antihyperuricemic agent; Y, years; 6Y–End Mean, mean value of pseudo-*R*^2^ from the 6th year to the study end; 6Y–End Change, percentage of change of pseudo-*R*^2^ values from the 6th year to the study end.

## Supplementary Materials

The following are available online at https://www.mdpi.com/2075-4426/10/4/248/s1. The appendix provides further methodological detail and results for the main paper. Supplementary Figure S1. Patient selection flow chart. Supplementary Figure S2. Kaplan–Meier survival curves of the kidney prognosis stratified by HU6, HU7, and HU8 for the entire cohort (A, D, G), men (B, E, H), and women (C, F, I). Supplementary Table S1. Patient characteristics according to sex (cohort without antihyperuricemic agents: *n* = 122). Supplementary Table S2. Results of the multivariable analyses for the risk factors associated with disease progression (i.e., a ≥ 30% estimated glomerular filtration rate decrease or end-stage kidney disease) in men, in women, and in patients without antihyperuricemic agents.
