# Supplementary material for: Sex Differences in Time-Series Changes in Pseudo-R2 Values Regarding Hyperuricemia in Relation to the Kidney Prognosis
Source: J Pers Med. 2020 Nov 26;10(4):248. doi: 10.3390/jpm10040248 (PMC7711484; doi:10.3390/jpm10040248)
Supplement: Supplementary file 1 [file jpm-10-00248-s001.pdf]

Web appendix to "Sex Differences in Time Series Changes in Pseudo-R^2 Values Regarding Hyperuricemia in Relation to the Kidney Prognosis"

Hiroshi Kataoka [1,2,], Mamiko Ohara [3], Toshio Mochizuki [1,2,*], Kazuhiro Iwadoh [4], Yusuke Ushio [1], Keiko Kawachi [1], Kentaro Watanabe [1], Saki Watanabe [1], Taro Akihisa [1], Shiho Makabe [1], Shun Manabe [1], Masayo Sato [1], Naomi Iwasa [1], Rie Yoshida [1], Yukako Sawara [1], Norio Hanafusa [4], Ken Tsuchiya [4] and Kosaku Nitta [1]

1 Department of Nephrology, Tokyo Women's Medical University, Tokyo 162-8666, Japan
2 Department of Nephrology, Clinical Research Division for Polycystic Kidney Disease, Tokyo Women's Medical University, Tokyo 162-8666, Japan
3 Department of Nephrology, Kameda Medical Center, Chiba, 296-8602, Japan
4 Department of Blood Purification, Tokyo Women's Medical University, Tokyo 162-8666, Japan

This appendix provides further methodological detail and results for the main paper.

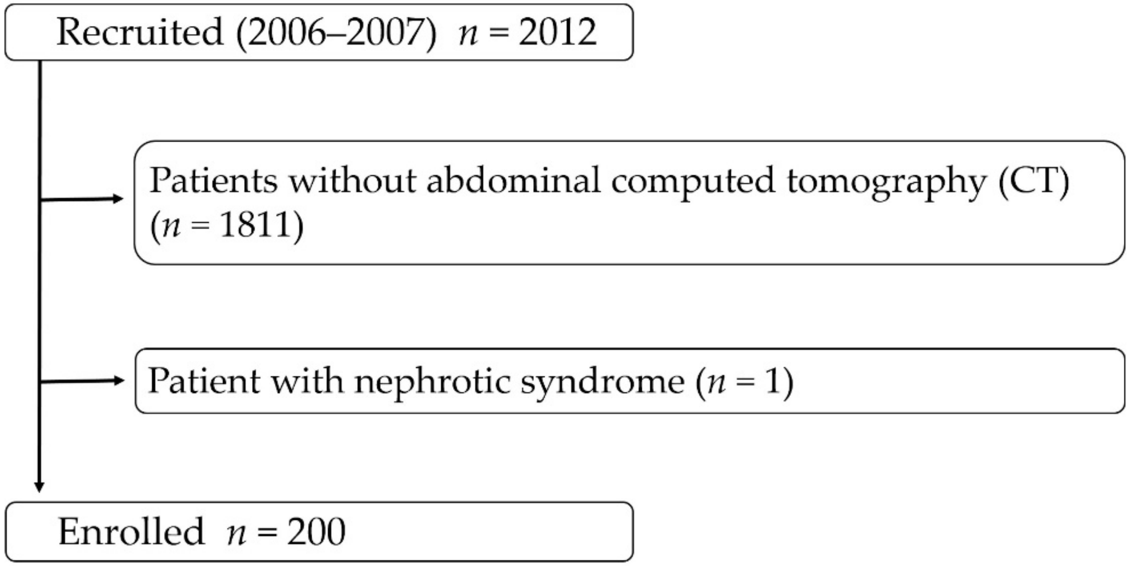

Supplementary Fig.S1. Patient selection flow chart. The patient selection flowchart is shown. Among 2012 screened patients, 1811 patients without abdominal computed tomography and 1 patient with nephrotic syndrome are excluded from the study. The remaining 200 patients are enrolled.

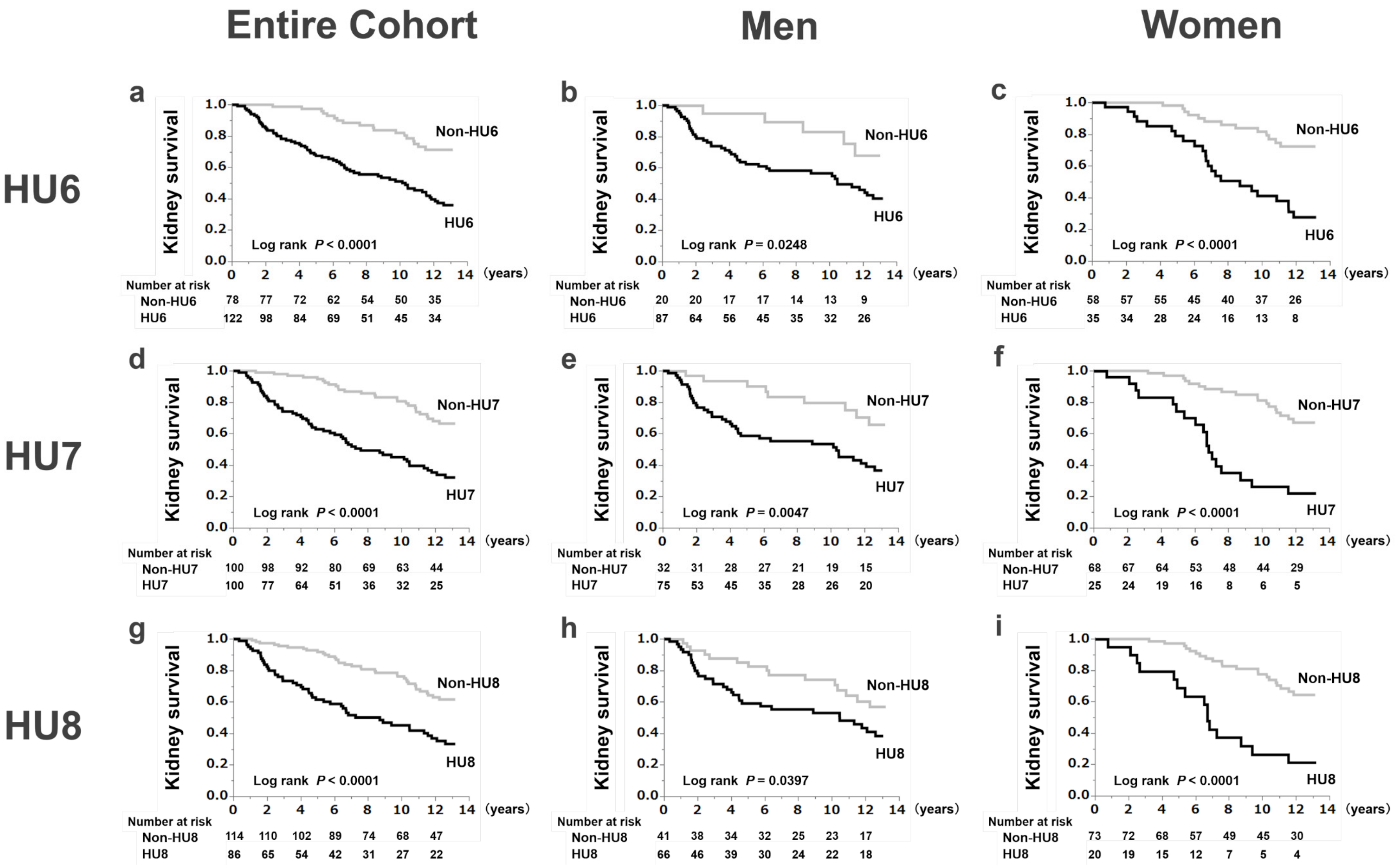

Supplementary Fig. S2. Kaplan–Meier survival curves of the kidney prognosis stratified by HU6, HU7, and HU8 for three cohorts: the entire cohort (a, d, g), men (b, e, h), and women (c, f, i). The kidney prognosis for patients meeting any of the hyperuricemia criteria is poor. Abbreviations: HU6, serum uric acid level ≥6.0 mg/dL or antihyperuricemic agent use; HU7, serum uric acid level ≥7.0 mg/dL or antihyperuricemic agent use; HU8, serum uric acid level ≥8.0 mg/dL or antihyperuricemic agent use; *P*, calculated probability.

Supplementary Table S1. Patient characteristics according to sex (Cohort without antihyperuricemic agents: *n* = 122).

Variables	Entire Cohort	Men	Women	*P*-Value
	n = 122	*n* = 47	*n* = 75	
Clinical and Laboratory Findings				
Age (years)	58.8 ± 13.7 [122]	59.3 ± 14.5	58.5 ± 13.3	0.7494
Sex (Men; %)	47 (38.5) [122]	47 (100.0)	0 (0.0)	<0.0001
MBP (mmHg)	92.4 ± 6.5[122]	94.1 ± 6.1	91.4 ± 6.5	0.0263
BMI (kg/m^2)	23.8 ± 4.1 [122]	24.3 ± 3.5	23.5 ± 4.5	0.3004
Visceral fat area (cm^2)	115.7 ± 61.5 [122]	142.1 ± 69.6	99.1 ± 49.5	0.0001
Visceral fat area 100 cm^2 (vs. no)	68 (55.7) [122]	33 (70.2)	35 (46.7)	0.0108
eGFR (mL/min/1.73m^2)	63.7 ± 19.8 [122]	59.8 ± 22.0	66.1 ± 18.0	0.0910
Uric Acid (mg/dL)	5.47 ± 1.55 [121]	6.35 ± 1.31	4.91 ± 1.43	<0.0001
UACR (mg/g Cre)	45.7 (19.2–133.8) [122]	40.5 (18.6–408.6)	46.7 (19.4–110.2)	0.2124
Primary cause of CKD				
Diabetic nephropathy (%)	11 (9.0) [122]	5 (10.6)	6 (8.0)	0.7477
Chronic glomerulonephritis (%)	69 (56.6) [122]	22 (46.8)	47 (62.7)	0.0855
Nephrosclerosis (%)	18 (14.8) [122]	13 (27.7)	5 (6.7)	0.0029
Others (%)	24 (19.7) [122]	7 (14.9)	17 (22.7)	0.3542
Concomitant drugs				
Antihypertensive agents (%)	76 (62.3) [122]	33 (70.2)	43 (57.3)	0.1532
ARB and or ACEI	59 (48.4) [122]	29 (61.7)	30 (40.0)	0.0196
CCB	31 (25.4) [122]	10 (21.3)	21 (28.0)	0.4065
Antidiabetic agents (%)	17 (13.9) [122]	10 (21.3)	7 (9.3)	0.1047
Corticosteroids (%)	19 (15.6) [122]	9 (19.2)	10 (13.3)	0.4459
Immunosuppressants (%)	6 (4.9) [122]	3 (6.4)	3 (4.0)	0.6749
Diuretics (%)	19 (15.6) [122]	6 (12.8)	13 (17.3)	0.6117
Comorbidities				
Hypertension (%)	76 (62.3) [122]	33 (70.2)	43 (57.3)	0.1532
HU6 (%)	44 (36.1) [122]	27 (57.5)	17 (22.7)	<0.0001
HU7 (%)	23 (18.9) [122]	16 (34.0)	7 (9.3)	0.0015
HU8 (%)	9 (7.4) [122]	7 (14.9)	2 (2.7)	0.0267
Hypertriglyceridemia (%)	69 (56.6) [122]	27 (57.5)	42 (56.0)	0.8753
Hypercholesterolemia (%)	73 (59.8) [122]	24 (51.1)	49 (65.3)	0.1177
Low HDL cholesterol (%)	51 (41.8) [122]	21 (44.7)	30 (40.0)	0.6100
Hyperglycemia (%)	41 (33.6) [122]	22 (46.8)	19 (25.3)	0.0145

Continuous variables are expressed as means and standard deviations or as medians (quartile 1–quartile 3). Categorical variables are expressed as n (%). Values of non-missing data are shown in brackets. Abbreviations: *P*, calculated probability; MBP, mean blood pressure; BMI, body mass index; eGFR, estimated glomerular filtration rate; UACR, urine albumin-to-creatinine ratio; Cre, creatinine; CKD, chronic kidney disease; ARB, angiotensin II receptor blocker; ACEI, angiotensin-converting enzyme inhibitor; CCB, calcium-channel blocker; HU6, serum uric acid level ≥6.0 mg/dL or antihyperuricemic agent use; HU7, serum uric acid level ≥7.0 mg/dL or antihyperuricemic agent use; HU8, serum uric acid level ≥8.0 mg/dL or antihyperuricemic agent use; HU (Drug), antihyperuricemic agent use; HDL, high-density lipoprotein.

Supplementary Table S2. Results of the multivariable analyses for the risk factors associated with disease progression (i.e., a ≥30% estimated glomerular filtration rate decline or end-stage kidney disease) in men, in women, and in patients without antihyperuricemic agents.

Variables in three sub-cohort	Multivariable Analysis for HU6		Multivariable Analysis for HU7		Multivariable Analysis for HU8	
	Hazard Ratio (95% CI)	*P*-Value	Hazard Ratio (95% CI)	*P*-Value	Hazard Ratio (95% CI)	*P*-Value
**Men (*n* = 107)**						
eGFR (10 mL/min/1.73 m^2 increments)	0.68 (0.57–0.81)	<0.0001	0.69 (0.58–0.82)	<0.0001	0.68 (0.56–0.81)	<0.0001
UACR (10 mg/g Cre increments)	1.00 (1.00–1.01)	0.0048	1.01 (1.00–1.01)	0.0064	1.00 (1.00–1.01)	0.0041
HU6 (vs. no)	1.49 (0.61–4.43)	0.4016	-	-	-	-
HU7 (vs. no)	-	-	1.48 (0.69–3.45)	0.3239	-	-
HU8 (vs. no)	-	-	-	-	1.07 (0.56–2.13)	0.8436
Hyperglycemia (vs. no)	1.60 (0.89–2.92)	0.1183	1.67 (0.91–3.07)	0.0957	1.60 (0.87–2.94)	0.1290
**Women (*n* = 93)**						
eGFR (10 mL/min/1.73 m^2 increments)	0.69 (0.56–0.86)	0.0007	0.70 (0.56–0.86)	0.0009	0.68 (0.55–0.85)	0.0006
UACR (10 mg/g Cre increments)	1.01 (1.00–1.01)	0.1201	1.01 (1.00–1.01)	0.1022	1.01 (1.00–1.01)	0.1451
HU6 (vs. no)	2.11 (0.96–4.75)	0.0628	-	-	-	-
HU7 (vs. no)	-	-	2.18 (1.01–4.69)	0.0467	-	-
HU8 (vs. no)	-	-	-	-	1.73 (0.77–3.84)	0.1808
Hyperglycemia (vs. no)	2.44 (1.08–5.18)	0.0326	2.27 (1.07–4.84)	0.0332	2.16 (0.98–4.47)	0.0571
**Without antihyperuricemic agents (*n* = 122)**						
eGFR (10 mL/min/1.73 m^2 increments)	0.84 (0.70–1.00)	0.0534	0.84 (0.69–1.00)	0.0529	0.84 (0.69–1.01)	0.0629
UACR (10 mg/g Cre increments)	1.01 (1.01–1.02)	<0.0001	1.01 (1.00–1.01)	0.0006	1.01 (1.01–1.02)	<0.0001
HU6 (vs. no)	1.91 (0.98–3.72)	0.0558	-	-	-	-
HU7 (vs. no)	-	-	2.23 (1.03–4.63)	0.0416	-	-
HU8 (vs. no)	-	-	-	-	2.74 (0.86–7.37)	0.0850
Hyperglycemia (vs. no)	1.29 (0.63–2.54)	0.4720	1.32 (0.64–2.62)	0.4380	1.28 (0.62–2.54)	0.5005

Variables with a *P*-Value < 0.05 in Table 2 and hyperuricemia were included in the multivariable model. Abbreviations: CI, confidence interval; *P*, calculated probability; eGFR, estimated glomerular filtration rate; UACR, urine albumin-to-creatinine ratio; HU6, serum uric acid level ≥6.0 mg/dL or antihyperuricemic agent use; HU7, serum uric acid level ≥7.0 mg/dL or antihyperuricemic agent use; HU8, serum uric acid level ≥8.0 mg/dL or antihyperuricemic agent use.
